# Clinical outcomes and biomarker exploration of first-line PD-1 inhibitors plus chemotherapy in patients with low PD-L1-expressing of gastric or gastroesophageal junction adenocarcinoma

**DOI:** 10.1007/s00262-024-03721-6

**Published:** 2024-06-04

**Authors:** Yu-Ting Sun, Shi-Xun Lu, Ming-Yu Lai, Xia Yang, Wen-Long Guan, Li-Qiong Yang, Yu-Hong Li, Feng-Hua Wang, Da-Jun Yang, Miao-Zhen Qiu

**Affiliations:** 1 Department of Medical Oncology, Sun Yat-sen University Cancer Center, State Key Laboratory of Oncology in South China, Collaborative Innovation Center for Cancer Medicine, Sun Yat-sen University, Guangzhou, 510060 People’s Republic of China; 2 Department of Pathology, Sun Yat-sen University Cancer Center, State Key Laboratory of Oncology in South China, Collaborative Innovation Center for Cancer Medicine, Sun Yat-sen University, Guangzhou, 510060 People’s Republic of China; 3 Department of Basic Research, Sun Yat-sen University Cancer Center, State Key Laboratory of Oncology in South China, Collaborative Innovation Center for Cancer Medicine, Sun Yat-sen University, Guangzhou, 510060 People’s Republic of China; 4 Department of Medical Oncology, National Cancer Center/National Clinical Research Center for Cancer/Cancer Hospital, Chinese Academy of Medical Sciences and Peking Union Medical College, Beijing, 100021 People’s Republic of China

**Keywords:** PD-1 inhibitors, Low PD-L1 expression, Biomarkers, Gastric cancer, Chemotherapy

## Abstract

**Background:**

The beneficial effects of first-line programmed death-1 (PD-1) inhibitors plus chemotherapy in patients with low programmed death-ligand 1 (PD-L1)-expressing advanced gastric or gastroesophageal junction (G/GEJ) adenocarcinoma are controversial.

**Methods:**

We conducted a retrospective analysis of patients with G/GEJ adenocarcinoma who had undergone first-line treatment with PD-1 inhibitors plus chemotherapy between October 2017 and May 2022. The primary outcomes were objective response rate (ORR) and progression-free survival (PFS). SPSS software V27.0 was used for data analysis.

**Results:**

Of 345 enrolled patients, 290 had measurable lesions. The overall ORR was 59.3%. PD-L1 status was available in 171 patients, and 67.8% of them were considered as low PD-L1 expression level (combined positive score (CPS) < 5). Patients with PD-L1 CPS < 5 showed a lower response rate (51.1% vs 70.8%,* P* = 0.024) and a worse PFS (*P* = 0.009) compared to those with PD-L1 CPS ≥ 5. In the PD-L1 low-expression cohort, patients with non-diffuse type, GEJ cancer, synchronous metastasis, distant lymph node metastasis, liver metastasis, non-peritoneal metastasis, and HER2 positive were significantly associated with higher response rates to PD-1 inhibitors plus chemotherapy (*P* < 0.05). The presence of peritoneal metastasis (*P* = 0.028) and diffuse type (*P* = 0.046) were identified as independent predictors of poor PFS in multivariate analysis of the PD-L1 CPS < 5 subgroup. When evaluated for correlation with overall survival (OS) in the PD-L1 low-expression subgroup, peritoneal metastasis was found to be the only independent prognostic factor of an increased risk of death (hazard ratio: 2.31, 95% CI 1.09–4.90; *P* = 0.029).

**Conclusions:**

PD-L1 CPS ≥ 5 is significantly associated with improved response and extended PFS in G/GEJ cancer patients treated with a combination of PD-1 inhibitors and chemotherapy. Specific subgroups within the low PD-L1-expressing population, such as those with non-diffuse-type tumors and without peritoneal metastases, may also benefit from immunotherapy combined with chemotherapy.

**Supplementary Information:**

The online version contains supplementary material available at 10.1007/s00262-024-03721-6.

## Introduction

Gastric cancer (GC) is the fifth most common malignancy and the fourth leading cause of cancer-related mortality [1]. The highest incidence area of GC is in Eastern Asia, while North America and Africa have the lowest incidence rates worldwide [2]. Histopathological examination is the gold standard to diagnose gastric cancer [3]. Owing to active early screening programs (such as endoscopy and *Helicobacter pylori* detection) and wide local excision, outcomes for GC patients have been improved [4]. However, many patients remain initially diagnosed at an advanced stage with a poor 5 years survival rate [5]. Cytotoxic chemotherapy is still the backbone treatment against advanced or metastatic gastric or gastroesophageal junction (G/GEJ) adenocarcinoma, with a median overall survival (OS) of 10–12 months [6]. Understanding the molecular profiling of G/GEJ cancer promotes the development of targeted therapies. For human epidermal receptor 2 (HER2) positive GC, the addition of trastuzumab to first-line chemotherapy improves survival [7]. Ramucirumab and apatinib also show positive results in previously treated advanced G/GEJ adenocarcinoma [8, 9]. However, cytotoxic chemotherapy and targeted agents still have limited efficacy in advanced or metastatic G/GEJ adenocarcinoma. New therapeutic options are required to improve patient survival.

Recently, immune checkpoint inhibitors (ICIs) have shown promising prospects in the treatment of several cancers [10]. Immune escape mediated by immune checkpoint proteins such as programmed death 1 (PD-1) and cytotoxic T-lymphocyte antigen 4 (CTLA-4) greatly contributes to the occurrence and development of tumors [11, 12]. The interaction of PD-1 and its ligand programmed death-ligand 1 (PD-L1) negatively regulates T-cell activity and creates an immunosuppressive environment [13, 14]. ICIs enhance the antitumor effect of T cells by blocking the interaction of immune checkpoint proteins with their ligands [15]. ICIs targeting the PD-1/PD-L1 pathway, such as nivolumab and sintilimab, have been proved to show good antitumor activity and safety in advanced G/GEJ adenocarcinoma [16, 17]. PD-L1 expression level has been validated as a predictive biomarker for the efficacy of ICIs across various tumor types. The combined positive score (CPS) and tumor proportion score (TPS) are the commonly used approaches to quantify PD-L1 expression. The CPS, defined as the ratio of PD-L1-stained cells (including tumor cells, lymphocytes, and macrophages) to the total number of viable tumor cells, is a more sensitive prognostic biomarker in GC [18]. A meta-analysis summarizing data from 12 clinical trials in G/GEJ cancer found that ICI monotherapy did not provide survival benefits in the PD-L1 negative population, and its efficacy improved incrementally with increasing CPS [19].

Preclinical investigations indicated that chemotherapy may boost immune response by tumor immunogenicity improvement, proinflammatory cytokines stimulation, immunosuppressive cell elimination, and reduced cytotoxic T-cell exhaustion [12, 20]. Owing to the potential antitumor synergism of immunotherapy and chemotherapy, their combinational efficacy in cancer treatment has been investigated. For advanced G/GEJ adenocarcinoma, data from five phase III randomized controlled trials were available; the effects of PD-1 inhibitor plus chemotherapy were compared with chemotherapy alone in the first-line setting [16, 21–24]. In the CheckMate 649 trial, superior OS and progression-free survival (PFS) benefits were reported for PD-L1 CPS ≥ 5, PD-L1 CPS ≥ 1, and all randomized patients who had received nivolumab plus chemotherapy; the benefits were enriched in patients with PD-L1 CPS ≥ 5 tumors [21]. Subgroup analysis by PD-L1 CPS did not inform in the CheckMate 649 trial, leaving the clinical efficacy of immunochemotherapy in low PD-L1-expressing G/GEJ adenocarcinoma unclear. Zhao et al. [25] used KMSubtraction to retrieve unreported PD-L1 subgroup data from the CheckMate 649 trial. The results suggested that adding nivolumab to chemotherapy in the low PD-L1-expressing subgroup (CPS 1–4; OS: hazard ratio [HR] 0.950, 95%CI 0.747–1.209) did not provide additional advantages [25]. Comparatively, the National Comprehensive Cancer Network (NCCN) guidelines only recommended anti-PD-1 antibody plus chemotherapy as the first-line treatment for advanced gastric cancer patients with PD-L1 CPS ≥ 5. Other studies, such as KEYNOTE-062 trial, patients with PD-L1 CPS ≥ 1 did not exhibit any survival benefits when receiving pembrolizumab combined with chemotherapy compared to chemotherapy alone [23]. The ORIENT-16 study verified that sintilimab plus chemotherapy was a better choice than chemotherapy, with longer survival in the PD-L1 CPS ≥ 5 cohort and in the whole group [16]. Therefore, it is debatable whether patients with low PD-L1-expressing G/GEJ adenocarcinoma can benefit from the anti-PD-1-chemotherapy combination.

Here, in this retrospective analysis, we investigated the clinical outcomes of first-line PD-1 inhibitors plus chemotherapy in PD-L1 CPS < 5 cohort and all enrolled G/GEJ adenocarcinoma patients. Further biomarker exploration for survival and efficacy prediction was also performed. These investigations would promote precise and cost-effective treatment with fewer adverse events.

## Methods

### Patients

A retrospective study was conducted by enrolling patients with G/GEJ adenocarcinoma who received PD-1 inhibitor plus chemotherapy as first-line treatment between October 2017 and May 2022 at Sun Yat-sen University Cancer Center (SYSUCC). The inclusion criteria for eligible patients were as follows: histologically confirmed G/GEJ adenocarcinoma, first-line therapy with anti-PD-1 antibody in combination with chemotherapy, and at least one treatment course with PD-1 inhibitor. The exclusion criteria included evidence of a second primary tumor and prior treatment with any ICIs. Some patients were previously recruited to the nivolumab and chemotherapy group of the CheckMate 649 trial.

### Ethics approval and consent to participate

This study was performed in accordance with the Declaration of Helsinki Protocols and was approved by the Ethics Committee of SYSUCC. As a retrospective study, the informed consent was waived.

### Clinical data extraction

Clinical data were extracted from medical records to identify potential prognostic factors, including age, sex, Eastern Cooperative Oncology Group Performance Status (ECOG PS), histological type, number and location of metastatic organs, and degree of differentiation. Baseline (within 1 week before starting ICIs and chemotherapy) and post-treatment (within 7 days of the first efficacy evaluation time) blood test results of lymphocyte, neutrophil, monocyte, and platelet counts were collected to calculate the neutrophil-to-lymphocyte ratio (NLR), monocyte-to-lymphocyte ratio (MLR), and platelet-to-lymphocyte ratio (PLR).

### Molecular biomarkers

Formalin-fixed paraffin-embedded tissue samples were used to assess the signals of molecular biomarkers including HER2, PD-L1, and EBV. HER2 status was detected by immunohistochemistry (IHC) staining with a monoclonal anti-HER2 primary antibody (4B5, VENTANA, USA) and fluorescence in situ hybridization (FISH) using a Jin Pujia GP HER2 probe kit (Beijing Jin Pujia Medical Technology Company Limited, Beijing, China). HER2 IHC staining was scored from 0 to 3+ , with 0 (no staining or faint membrane staining in < 10% of tumor cells), 1+ (weak membrane staining in ≥ 10% of tumor cells), 2+ (weak to moderate basolateral, lateral, or complete membrane staining in ≥ 10% of tumor cells), and 3+ (strong basolateral, lateral, or complete membrane staining in ≥ 10% of tumor cells). IHC 2+ with HER2 gene amplification confirmed by FISH and IHC 3+ were regarded as HER2 positive. HER2 gene amplification was defined as a HER2/chromosome enumeration probe 17 (CEP17) ratio ≥ 2.0 in tumor cells [26]. IHC for PD-L1 was performed using an anti-PD-L1 monoclonal antibody (E1L3N; cell signaling technology, USA). PD-L1 expression was evaluated using the combined positive score (CPS), defined as the number of PD-L1-stained cells (tumor cells, lymphocytes, and macrophages) divided by the total number of viable tumor cells, multiplied by 100. EBV status was determined using an EBV-encoded RNA (EBER) in situ hybridization kit (Beijing Zhongshan Jinqiao Biotechnology Company Limited, Beijing, China). Brown–yellow stained nuclei were reliable EBER-positive staining.

Data on MMR status, microsatellite instability (MSI) type, and tumor mutation burden (TMB) were directly collected from medical records, if available. Tumors expressing MLH1, MSH2, MSH6, and PMS2 were considered MMR-proficient (P-MMR), while those with a lack of expression of any of these were considered as MMR-deficient (D-MMR) tumors. Next-generation sequencing (NGS)-based assays were used to determine MSI types, including MSI-high (MSI-H), MSI-ambiguous, and microsatellite stable (MSS). TMB refers to the total number of somatic mutations in the coding area of the tumor genome as indels per megabase (Mb). Tissue TMB ≥ 10 mutations/Mb was defined as TMB-high.

### *Helicobacter pylori* infection

Serological examination was performed using the MP Diagnostics ASSURE *H. pylori* Rapid Test (MP Biomedicals Asia Pacific Pte Ltd, Singapore) to confirm *H. pylori* infection status. Archival serum samples collected within 1 week before the initial first-line treatment were acquired. Serum and buffer were added according to the operating procedure. The results were recorded after 15 min. Three bands were observed on the reaction board, of which band “A” was used for quality control, band “B” was considered as the current infection marker (CIM), and “C” was the test band. Current *H. pylori* infection was confirmed by the presence of “A,” “B,” and “C.” A negative result was defined as only the band “A” being visible. All test results were diagnosed by two investigators.

### Outcome evaluation

Tumor response was assessed according to the Response Evaluation Criteria in Solid Tumors (RECIST) V.1.1, by comparing images of patients with measurable lesions, every 6 weeks. Responders were defined as patients with the best overall response of complete response (CR) or partial response (PR). Non-responders included those with stable disease (SD) or progressive disease (PD). Objective response rate (ORR) was defined as the proportion of responders. Progression-free survival (PFS) was calculated from the date of initiation of ICIs plus chemotherapy to the date of disease progression, last follow-up, or death due to any cause. OS was defined as the period from the date of the first administration of ICIs plus chemotherapy until the last follow-up or death for any reason.

### Statistical analysis

For baseline characteristics, the Student’s *t *test was used to examine differences between two groups of quantitative data with normal distribution, while those with non-normal distribution utilized the Mann–Whitney *U* test. The chi-square test or Fisher’s exact test was applied to compare the qualitative variables. Comparative analysis for ORR was performed using the chi-square test or Fisher’s exact test, depending on the clinical and molecular characteristics. Survival curves were plotted using the Kaplan–Meier method and compared using the log-rank test. Univariate and multivariate Cox proportional hazard models were established to examine the relationship between potential biomarkers and survival outcomes by calculating HRs with 95% confidence intervals (CIs). The median was selected as the cutoff value for NLR, MLR, and PLR. A ratio above the cutoff value was defined as the high-value group. SPSS software V27.0 was used for data analysis, and graphs were plotted using GraphPad Prism software V9.1.1. All tests were two-sided, and *P* < 0.05 was considered statistically significant.

## Results

### Patient characteristics

**Fig. 1:**
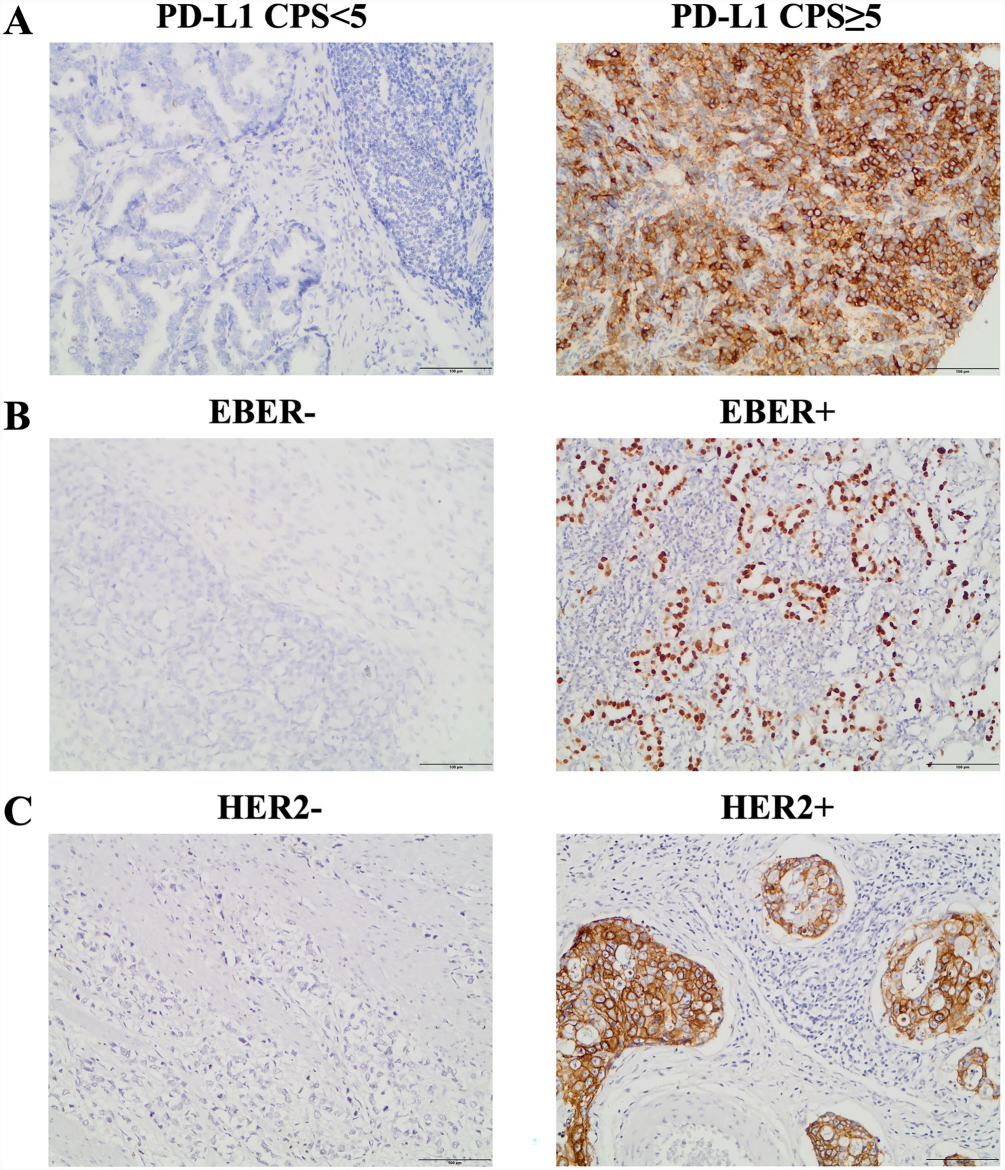
Representative immunohistochemistry staining images (20×) of the PD-L1 (**A**), EBER (**B**), and HER2 (**C**) in the enrolled patients. PD-L1, programmed death-ligand 1; CPS, combined positive score; EBER, EBV-encoded RNA; HER2, human epidermal growth factor receptor 2

A total of 345 patients were enrolled in this retrospective analysis, 290 of whom had measurable lesions. Supplementary Table S1 summarizes the baseline characteristics of the whole population. Representative immunohistochemistry staining images (20×) of the PD-L1, EBER, and HER2 are shown in Fig. 1. PD-L1 expression levels were assessed in 171 patients. 116 individuals were confirmed to have low PD-L1 expression levels (CPS < 5), with 94 of them having measurable lesions. The baseline features of patients with PD-L1 CPS < 5 and PD-L1 CPS ≥ 5 are compared and described in Supplementary Table S2. In PD-L1 CPS < 5 cohort, the median age was 57 (range 25–75) years, and 62.1% of the patients were male. The majority (98.3%) of patients with low PD-L1 expression had an ECOG PS of 0 or 1. The peritoneum was the most common metastatic site, accounting for 57.8% of the total, followed by the distant lymph node (48.3%) and the liver (34.5%). Of 57 *H. pylori* infection status evaluable low PD-L1-expressing patients, 35 (61.4%) were positive. HER2 positivity was confirmed in 21 (18.3%) of the 115 detectable patients, and 1 was EBV-positive (1.1%) of the 92 examined cases. MMR status were available for 97 patients; all of them had P-MMR tumors. One (3.7%) of the 33 patients had high TMB. When compared to the population with PD-L1 CPS ≥ 5, the cohort with PD-L1 CPS < 5 exhibited a lower rate of EBV positivity and lymph node metastasis occurrence, but showed an increased incidence of peritoneal metastasis.

### Efficacy and survival in the total population

The tumor response rates were evaluated in 290 patients with measurable lesions. The numbers of patients with the best overall responses as CR, PR, SD, and PD were 14 (4.8%), 158 (54.5%), 93 (32.1%), and 25 (8.6%), respectively. Therefore, the overall ORR was 59.3%. Supplementary Table S3 shows the association between clinicopathological features and ORR in all enrolled patients. Synchronous metastasis (63.3%, *P* = 0.004), non-diffuse type (68.5%, *P* = 0.001), male (63.8%,* P* = 0.033), liver metastasis (67.2%, *P* = 0.022), lymph node metastasis (66.1%, *P* = 0.002), HER2 positive (79.2%, *P* = 0.001), and PD-L1 CPS $\ge$ 5 (70.8%, *P* = 0.024) were clinicopathologic factors significantly associated with higher response rates to ICI plus chemotherapy. Patients with peritoneal metastasis showed poorer response rates than those without (41.9% vs 72.3%, *P* < 0.001).

**Fig. 2:**
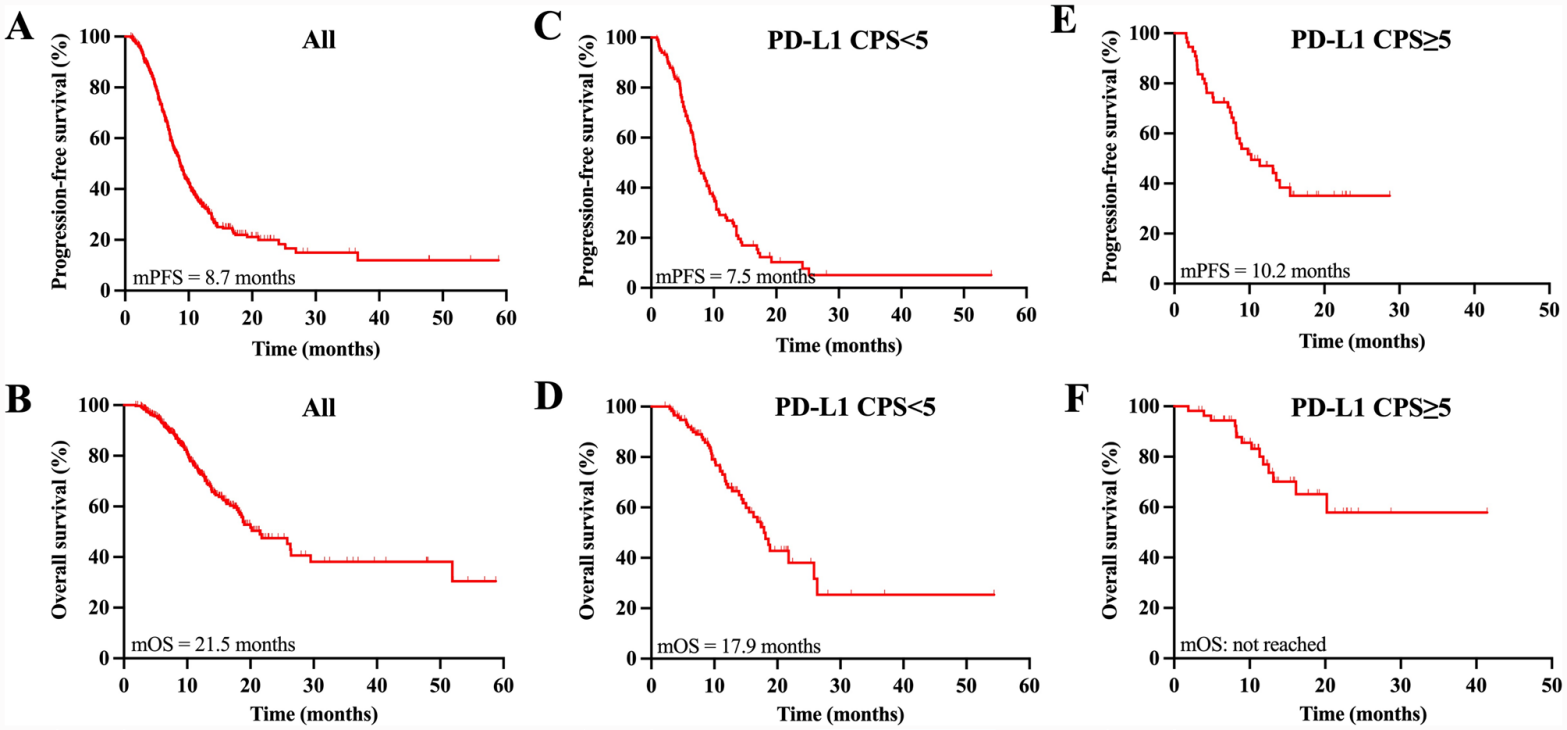
Progression-free survival and overall survival of the whole population (**A**, **B**), PD-L1 CPS < 5 subgroup (**C**, **D**), and PD-L1 CPS ≥ 5 subgroup (E, F). mOS, median overall survival. mPFS, median progression-free survival

The median PFS and OS were 8.7 months (95% CI, 7.8–9.5) and 21.5 months (95% CI, 16.5–26.4), respectively (Fig. 2A, B). Univariate and multivariate Cox analyses revealed that the presence of peritoneal metastasis (*P* = 0.018), PLR-high (*P* = 0.018), diffuse type (*P* = 0.018), HER2-negative status (*P* = 0.048), and PD-L1 CPS < 5 (*P* = 0.009) were identified as independent indicators of poor PFS (Supplementary Table S4). Peritoneal metastasis was the only significant predictor of an increased risk of death (HR 2.17, 95% CI 1.12–4.20; *P* = 0.022) (Supplementary Table S5).

### *Efficacy in patients with PD-L1 CPS* < *5*

The low PD-L1-expressing subgroup got a 51.1% response rate to PD-1 inhibitors plus chemotherapy. Next, we tried to determine what kinds of patients may benefit more from immunochemotherapy when the PD-L1 CPS < 5 (Table 1). Higher response rates were observed in patients with non-diffuse type than in those with diffuse type (70.6% vs 28.6%, *P* < 0.001) and synchronous metastasis than in those with metachronous metastasis (60.8% vs 15.0%, *P* < 0.001). GEJ cancer achieved an ORR of 100.0%, which was markedly higher than that of gastric cancer (45.2%;* P* = 0.001). Compared with patients without lymph node or liver metastasis, those with such conditions had significantly higher response rates. However, patients with peritoneal metastasis showed worse responses than those without metastasis (29.8% vs 72.3%, *P* < 0.001). Baseline NLR/MLR/PLR and *H. pylori* infection status were not predictive of ORR. HER2 was the sole molecular pathological factor associated with response. The ORRs for HER2-positive versus HER2-negative tumors were 80.0% and 43.8%, respectively (*P* = 0.004). Patients who possessed at least one of the following traits (non-diffuse type, HER2-positive status, and/or absence of peritoneal metastasis) had greater ORRs than those without (64.1% vs 23.1%, *P* < 0.001).

**Table 1:** Clinicopathologic features of responders and non-responders in the PD-L1 CPS $<$ 5 population

Characteristics		Responder *N* = 48	Non-responder *N* = 46	ORR (%)	*P* value
Age	$<$ 60	26	27	49.1	0.658
	$\ge$ 60	22	19	53.7	
Sex	Male	34	26	56.7	0.149
	Female	14	20	41.2	
BMI	$<$ 18.5	7	5	58.3	0.764
	18.5–23.9	31	29	51.7	
	$\ge$ 24	10	12	45.5	
Histology	Diffuse	10	25	28.6	< 0.001
	Non-diffuse	36	15	70.6	
Primary tumor location	Gastric cancer	38	46	45.2	0.001
	Gastroesophageal junction cancer	10	0	100.0	
Differentiation	High or middle differentiation	12	8	60.0	0.368
	Low differentiation	36	38	48.6	
Disease status	Synchronous metastasis	45	29	60.8	< 0.001
	Metachronous metastasis	3	17	15.0	
ECOG PS	0	38	30	55.9	0.131
	$\ge$ 1	10	16	38.5	
Site of metastasis					
Peritoneum	Yes	14	33	29.8	< 0.001
	No	34	13	72.3	
Liver	Yes	28	12	70.0	0.002
	No	20	34	37.0	
Lymph node	Yes	34	22	60.7	0.023
	No	14	24	36.8	
Ovary	Yes	4	9	30.8	0.332
	No	10	11	47.6	
Number of metastatic sites	$\le$ 1	15	19	44.1	0.311
	$\ge$ 2	33	27	55.0	
HER2	Negative	32	41	43.8	0.004
	Positive	16	4	80.0	
TMB	$<$ 10	9	17	34.6	0.370
	$\ge$ 10	1	0	100.0	
Baseline NLR	$<$ 3	21	28	42.9	0.097
	$\ge$ 3	27	18	60.0	
Baseline MLR	$<$ 0.31	26	27	49.1	0.658
	$\ge$ 0.31	22	19	53.7	
Baseline PLR	$<$ 188	24	22	52.2	0.833
	$\ge$ 188	24	24	50.0	
*H. pylori* infection	Yes	19	10	65.5	0.155
	No	8	10	44.4	
Composite variable	Non-diffuse type or HER2 (+) or without peritoneal metastasis	41	23	64.1	< 0.001
	Diffuse type and HER2 (−) and peritoneal metastasis	6	20	23.1	

ORR, objective response rate; ECOG PS, Eastern Cooperative Oncology Group performance status; BMI, body mass index; PD-L1, programmed death-ligand 1; CPS, combined positive score; HER2, human epidermal growth factor receptor 2; TMB, tumor mutational burden; NLR, neutrophil-to-lymphocyte ratio; MLR, monocyte-to-lymphocyte ratio; PLR, platelet-to-lymphocyte ratio

### *Progression-free survival in patients with PD-L1 CPS* < *5*

**Fig. 3:**
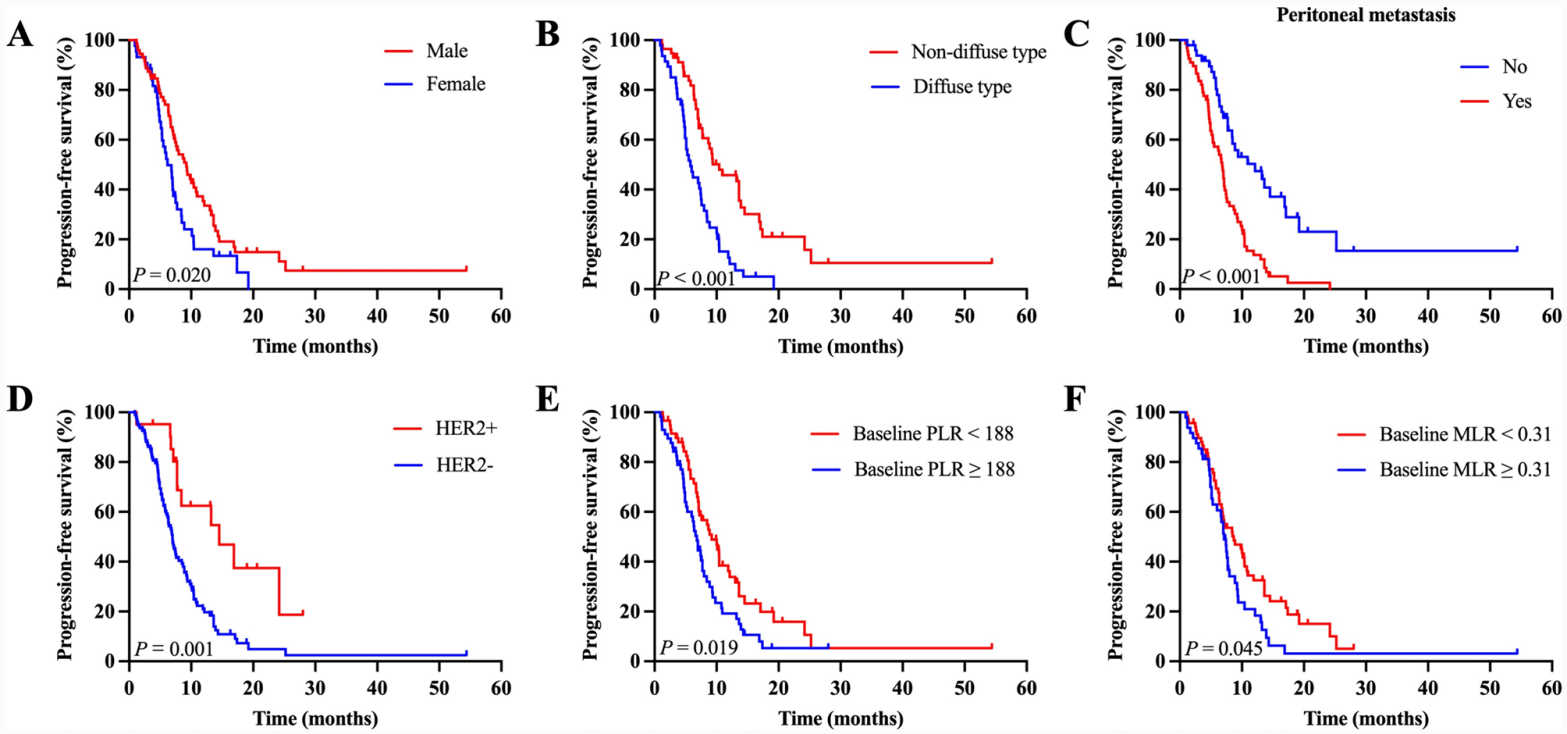
Kaplan–Meier curves of progression-free survival according to sex (**A**), histology (**B**), peritoneal metastasis (**C**), HER2 (**D**), baseline PLR (**E**), and baseline MLR (**F**) in the PD-L1 CPS < 5 population. PD-L1, programmed death-ligand 1; CPS, combined positive score; HER2, human epidermal growth factor receptor 2; PLR, platelet-to-lymphocyte ratio; MLR, monocyte-to-lymphocyte ratio

The median PFS in patients with PD-L1 low expression was 7.5 months (95% CI, 6.2–8.7) (Fig. 2C). Kaplan–Meier curves for PFS according to clinicopathological characteristics are shown in Fig. 3. Significantly inferior PFS was observed in patients with diffuse-type tumors in comparison with non-diffuse-type tumors (median 5.77 vs 10.40 months, *P* < 0.001), female compared to male (median 6.17 vs 9.23 months, *P* = 0.020), PLR-high than PLR-low (median 6.83 vs 9.23 months, *P* = 0.019), and MLR-high instead of MLR-low (median 7.10 vs 8.67 months, *P* = 0.045). PFS was shortened if peritoneal metastasis existed (median 6.83 vs 12.10 months, *P* < 0.001). As for molecular factors, HER2 positive (median 14.50 vs 6.97 months, *P* = 0.001) was found to predict longer PFS. There were no significant differences among the different TMB levels and *H. pylori* infection status.

Univariate Cox analysis showed that peritoneal metastasis, sex, PLR, MLR, histology, and HER2 expression level were significantly associated with PFS (Table 2). Two independent predictors of poor PFS were found by multivariate analysis: the presence of peritoneal metastasis (HR 1.80, 95% CI 1.07–3.04; *P* = 0.028) and diffuse type (HR 1.72, 95% CI 1.01–2.91; *P* = 0.046).

**Table 2:** Univariate and multivariate analyses for progression-free survival in the PD-L1 CPS $<$ 5 population

Variables		Univariate analysis		Multivariate analysis	
		HR (95% CI)	*P* value	HR (95% CI)	*P* Value
Age	$\ge$ 60 versus $<$ 60	0.77 (0.51–1.18)	0.236	–	–
Sex	Male versus female	0.61 (0.40–0.93)	0.021	0.86 (0.51–1.46)	0.583
BMI	$<$ 18.5	Reference			
	18.5–23.9	1.02 (0.54–1.90)	0.961	–	–
	$\ge$ 24	0.86 (0.42–1.75)	0.675	–	–
ECOG PS	0 versus $\ge$ 1	1.05 (0.67–1.66)	0.829	–	–
Primary tumor location	GEJC versus GC	0.45 (0.18–1.11)	0.084	–	–
Histology	Diffuse versus non-diffuse	2.53 (1.61–3.98)	< 0.001	1.72 (1.01–2.91)	0.046
Differentiation	High or middle differentiation versus low differentiation	0.85 (0.47–1.52)	0.575	–	–
Disease status	Synchronous metastasis versus metachronous metastasis	0.73 (0.44–1.22)	0.233	–	–
Number of metastatic sites	$\le$ 1 versus $\ge$ 2	0.94 (0.62–1.42)	0.750	–	–
Site of metastasis	Peritoneum	2.66 (1.68–4.20)	< 0.001	1.80 (1.07–3.04)	0.028
	Lymph node	0.86 (0.57,1.31)	0.483	–	–
	Liver	0.74 (0.47–1.16)	0.189	–	–
	Ovary	0.53 (0.26–1.07)	0.076	–	–
HER2	Positive versus negative	0.36 (0.19–0.67)	0.001	0.50 (0.24–1.04)	0.062
TMB	$\ge$ 10 versus $<$ 10	0.03 (0.00–8.53)	0.230	–	–
Baseline NLR	$\ge$ 3 versus $<$ 3	1.40 (0.93–2.13)	0.110	–	–
Baseline MLR	$\ge$ 0.31 versus $<$ 0.31	1.53 (1.01–2.34)	0.047	1.34 (0.78–2.29)	0.295
Baseline PLR	$\ge$ 188 versus $<$ 188	1.63 (1.08–2.47)	0.021	1.67 (0.99–2.82)	0.055
*H. pylori* infection	Yes versus no	0.99 (0.55–1.75)	0.958	–	–

HR, hazard ratio; CI, confidence interval; ECOG PS, Eastern Cooperative Oncology Group performance status; BMI, body mass index; GC, gastric cancer; GEJC, gastroesophageal junction cancer; PD-L1, programmed death-ligand 1; CPS, combined positive score; HER2, human epidermal growth factor receptor 2; TMB, tumor mutational burden; NLR, neutrophil-to-lymphocyte ratio; MLR, monocyte-to-lymphocyte ratio; PLR, platelet-to-lymphocyte ratio

### *Overall survival in patients with PD-L1 CPS* < *5-*

**Fig. 4:**
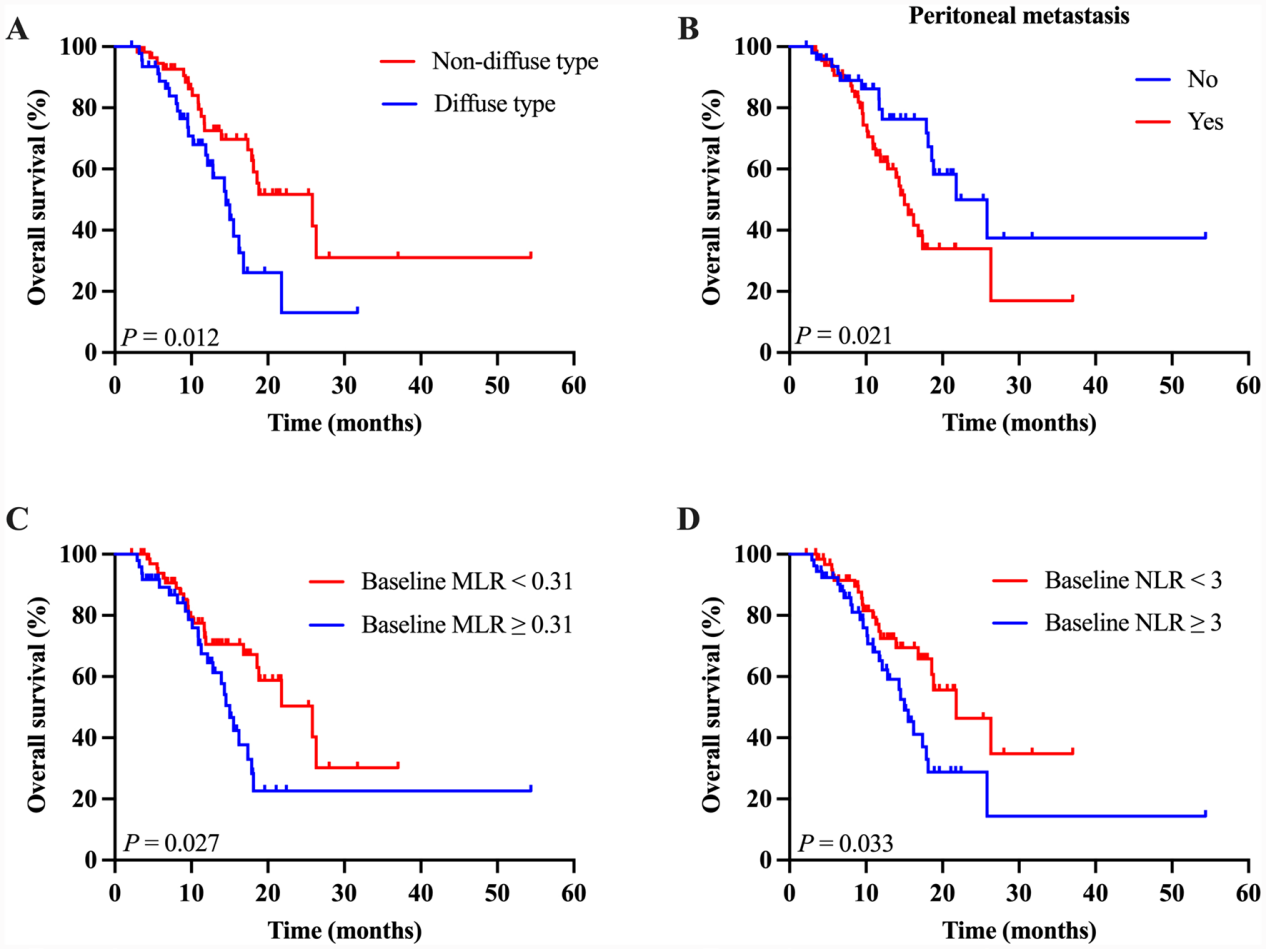
Kaplan–Meier curves of overall survival according to histology (**A**), peritoneal metastasis (**B)**, baseline MLR (**C**), and baseline NLR (**D**) in the PD-L1 CPS < 5 population. NLR, neutrophil-to-lymphocyte ratio; MLR, monocyte-to-lymphocyte ratio

The median OS reached 17.9 months (95% CI, 15.5–20.2) in this study (Fig. 2D). Enhanced OS benefit was revealed in patients with non-diffuse type than diffuse type (median 25.83 vs 14.50 months, *P* = 0.012), low baseline NLR than high baseline NLR (median 21.77 vs 15.00 months, *P* = 0.033), and baseline MLR-low than baseline MLR-high (median 25.83 vs 15.00 months, *P* = 0.027) (Fig. 4). The development of peritoneal metastasis was related to worse OS compared to those who did not (median 15.00 vs 21.77 months, *P* = 0.021). No significant differences were observed between the groups stratified by HER2 expression (*P* = 0.051); however, HER2-positive patients got a median survival of 26.33 months, whereas HER2-negative patients only had a median survival of 16.20 months. After univariate and multivariate Cox analyses (Table 3), peritoneal metastasis was the only independent prognostic factor associated with a shorter survival period (HR 2.31, 95% CI 1.09–4.90; *P* = 0.029).

**Table 3:** Univariate and multivariate analyses for overall survival in the PD-L1 CPS $<$ 5 population

Variables		Univariate analysis		Multivariate analysis	
		HR (95% CI)	*P* value	HR (95% CI)	*P* Value
Age	$\ge$ 60 versus $<$ 60	1.08 (0.60–1.95)	0.803	–	–
Sex	Male versus female	1.20 (0.65–2.23)	0.560	–	–
BMI	$<$ 18.5	Reference			
	18.5–23.9	0.77 (0.33–1.78)	0.541	–	–
	$\ge$ 24	0.72 (0.28–1.89)	0.505	–	–
ECOG PS	0 versus $\ge$ 1	0.72 (0.39–1.31)	0.280	–	–
Primary tumor location	GEJC versus GC	0.21 (0.03–1.49)	0.117	–	–
Histology	Diffuse versus non-diffuse	2.16 (1.17–3.97)	0.013	1.48 (0.74–2.95)	0.269
Differentiation	High or middle differentiation versus low differentiation	0.60 (0.24–1.51)	0.275	–	–
Disease status	Synchronous metastasis versus metachronous metastasis	0.61 (0.32–1.19)	0.146	–	–
Number of metastatic sites	$\le$ 1 versus $\ge$ 2	1.21 (0.68–2.17)	0.523	–	–
Site of metastasis	Peritoneum	2.08 (1.10–3.92)	0.024	2.31 (1.09–4.90)	0.029
	Lymph node	0.83 (0.46,1.49)	0.531	–	–
	Liver	0.64 (0.32–1.27)	0.204	–	–
	Ovary	0.39 (0.12–1.23)	0.107	–	–
HER2	Positive versus negative	0.41 (0.16–1.04)	0.059	0.62 (0.22–1.70)	0.348
TMB	$\ge$ 10 versus$<$ 10	0.04 (0.00–316.80)	0.490	–	–
Baseline NLR	$\ge$ 3 versus $<$ 3	1.87 (1.04–3.36)	0.036	1.92 (0.80–4.61)	0.144
Baseline MLR	$\ge$ 0.31 versus $<$ 0.31	1.92 (1.07–3.45)	0.029	1.19 (0.50–2.83)	0.694
Baseline PLR	$\ge$ 188 versus $<$ 188	1.22 (0.68–2.19)	0.505	–	–
*H. pylori* infection	Yes versus no	0.62 (0.30–1.26)	0.185	–	–

HR, hazard ratio; CI, confidence interval; ECOG PS, Eastern Cooperative Oncology Group performance status; BMI, body mass index; GC, gastric cancer; GEJC, gastroesophageal junction cancer; PD-L1, programmed death-ligand 1; CPS, combined positive score; HER2, human epidermal growth factor receptor 2; TMB, tumor mutational burden; NLR, neutrophil-to-lymphocyte ratio; MLR, monocyte-to-lymphocyte ratio; PLR, platelet-to-lymphocyte ratio

## Discussion

Immune evasion is a hallmark of tumorigenesis and development [27]. In particular, PD-1/PD-L1 pathway mediated immunosuppression has become a focus point of interest [28]. Elevated expression of PD-L1 on tumor cells can promote T-cell anergy and apoptosis, resulting in reduced tumor-specific immunity and tumor progression [29]. The advent of PD-1/PD-L1 inhibitors is a revolutionary breakthrough in cancer therapy, with promising application prospects in a variety of tumors. In G/GEJ adenocarcinoma, the indications for ICIs targeting the PD-1/PD-L1 pathway have gradually progressed from third-line to first-line treatment, establishing their important role in advanced systemic therapy. PD-1 inhibitor plus chemotherapy has been approved as the standard first-line treatment for advanced G/GEJ adenocarcinomas, with response rates fluctuating between 50 and 65% [16, 21]. However, the NCCN guidelines recommended this regimen for the PD-L1 CPS ≥ 5 population and the FDA for the entire population. There is ongoing debate about its efficacy in patients with low PD-L1 expression. Several clinical trials have performed subgroup analyses based on PD-L1 CPS cutoff values of 1, 5, and 10 in treatment-naive advanced G/GEJ adenocarcinomas; nevertheless, inconsistent outcomes were observed [21, 23]. In this study, we first investigated the efficacy and survival of immunochemotherapy in the total population and confirmed the benefit superiority of PD-L1 CPS ≥ 5 population compared to PD-L1 CPS < 5. In patients with low PD-L1 expression, we still found that 51.1% of patients responded to the combination of PD-1 inhibitors and chemotherapy. Biomarker exploration revealed characteristics of potential beneficiaries in PD-L1 low-expression cohort.

Peritoneal metastasis is a common metastatic pattern in advanced G/GEJ adenocarcinomas, suggesting a poor prognosis. The association between peritoneal metastasis and the benefits of immunochemotherapy in GC has not been clearly explained. Worse clinical outcomes of ICI monotherapy in patients with peritoneal disseminated GC have been observed in some retrospective studies, as well as in non-small cell lung cancer (NSCLC) [30–32]. In a post hoc analysis of ATTRACTION-2, peritoneal metastasis negatively affected the therapeutic effect of nivolumab in GC salvage therapy [33]. Similarly, in this study, peritoneal metastasis was an independent risk factor for poor PFS and OS in both the overall population and the PD-L1 low-expression group receiving PD-1 inhibitors plus chemotherapy as first-line treatment. Possible reasons for this observation are the lower PD-L1 positivity and highly invasive behavior of GC with peritoneal dissemination [34, 35]. Liver metastasis is believed to restrain immunotherapy efficacy in some cancers, such as NSCLC and melanoma [36, 37]; our study revealed it to be a positive predictor of immunochemotherapy in gastric cancer.

GC can be divided into diffuse, intestinal, and mixed types, according to the Lauren classification system [38]. Diffuse-type GC generally exhibits more aggressive characteristics and a poorer prognosis than intestinal-type GC [39, 40]. The major classification of diffuse-type GC is the “genome stable type,” which is unresponsive to checkpoint inhibitors [41]. Our findings suggest worse efficacy of PD-1 inhibitor plus chemotherapy in diffuse-type G/GEJ cancer than in non-diffuse type across the entire cohort and PD-L1 CPS < 5 subgroup. Moreover, diffuse-type GC is associated with a higher risk of peritoneal metastasis [42].

PD-L1 expression, HER2 positive, EBV infection, D-MMR/MSI-H, and TMB-high are well-known biomarkers to predict response from PD-1 inhibitor single agents in the third-line setting [43]; however, their value in predicting response to immunochemotherapy is unknown. The relationship between PD-L1 expression levels and ICI efficacy has been demonstrated in several cancers [44, 45]. In our analysis, PD-L1 CPS ≥ 5 was significantly associated with a better response and longer PFS to PD-1 inhibitor plus chemotherapy in G/GEJ cancer patients, which was in accordance with the findings of CheckMate 649 and ORIENT-16 [16, 21]. Some members of the low PD-L1-expressing population, such as those with non-diffuse-type tumors and without peritoneal metastases, can benefit from immunotherapy coupled with chemotherapy. Therefore, we cannot give up the opportunity to undergo immunotherapy simply based on the PD-L1 CPS < 5.

The combination of ICIs with anti-HER2 therapy has synergistic effects on HER2-positive tumors [46, 47]. The results of two phase II clinical trials in patients with HER-positive advanced G/GEJ cancer showed that the combination of pembrolizumab, trastuzumab, and chemotherapy as the first-line treatment strategy exhibited ORRs of 91.0% [48] and 77.0% [49], respectively, and the phase III KEYNOTE-811 trial achieved a 74.4% response rate [50]. Similarly, we observed an 80.0% response rate in HER2-positive patients of the PD-L1 CPS < 5 subgroup, which was significantly higher than that observed in HER2-negative patients. In all enrolled participants, considerably prolonged PFS was seen in the HER2-positive group. Although the median time to progression of HER2-positive patients in the PD-L1 CPS < 5 population was twice that of HER2-negative patients, this difference was not statistically significant.

Interestingly, our analysis showed that low PD-L1-expressing patients exhibiting at least one of the following characteristics: non-diffuse type, HER2 positive, and/or absence of peritoneal metastasis, had higher response rates than those without any of these factors. Taken together, these results suggest that multifactorial combination is a more precise biomarker for efficacy prediction of PD-1 inhibitors plus chemotherapy.

Inflammatory markers in peripheral blood, including NLR, PLR, and MLR, have been reported as predictors of immunotherapy efficacy in different malignancies [51, 52]. A previous study found a correlation between baseline PLR and PFS in patients with advanced G/GEJ cancer receiving ICIs plus chemotherapy as first-line therapy [53]. Similarly, we found that high baseline PLR was an independent risk factor for PFS in the whole population. *Helicobacter pylori* seropositivity has been identified as a risk factor for poor immunotherapy response in NSCLC [54]. In gastric cancer, we failed to prove a relationship between *H. pylori* infection and the clinical outcomes of immunochemotherapy.

However, this study had some limitations. First, this was a retrospective study with a limited sample size based on data from a single institution. Second, information on PD-L1, HER2, MMR, EBV, *H. pylori* infection, and TMB was not available for some of the enrolled patients. Further prospective investigations are required to validate our findings.

Our research is a supplement to previously reported phase 3 trials exploring immunotherapy coupled with chemotherapy in the first-line setting of gastric cancer. Worse clinical outcomes to PD-1 inhibitors plus chemotherapy were observed in patients with low PD-L1-expressing G/GEJ adenocarcinoma compared with PD-L1 CPS ≥ 5 cohort. Meanwhile, we identified several clinical and molecular biomarkers that can be used alone or in combination with predict efficacy, especially in the PD-L1 CPS < 5 cohort. Peritoneal metastasis and Lauren classification can be used by clinicians to predict the efficacy of chemotherapy plus PD-1 blockade in patients with PD-L1 CPS $<$ 5. New and precise predictive biomarkers are still required for G/GEJ adenocarcinoma patients receiving immunochemotherapy.

### Supplementary Information

Below is the link to the electronic supplementary material.

Supplementary file1 (DOCX 25 KB)

Supplementary file2 (DOCX 26 KB)

Supplementary file3 (DOCX 29 KB)

Supplementary file3 (DOCX 26 KB)

Supplementary file3 (DOCX 28 KB)

## Data Availability

Data are available upon reasonable request.

## References

[1] Smyth EC, Nilsson M, Grabsch HI, van Grieken NC, Lordick F (2020) Gastric cancer. Lancet 396(10251):635–648 32861308 10.1016/S0140-6736(20)31288-5

[2] Guan WL, He Y, Xu RH (2023) Gastric cancer treatment: recent progress and future perspectives. J Hematol Oncol 16(1):57 37245017 10.1186/s13045-023-01451-3 PMC10225110

[3] Wang FH, Zhang XT, Li YF, Tang L, Qu XJ, Ying JE, Zhang J, Sun LY, Lin RB, Qiu H et al (2021) The chinese society of clinical oncology (CSCO): clinical guidelines for the diagnosis and treatment of gastric cancer 2021. Cancer Commun (Lond) 41(8):747–795 34197702 10.1002/cac2.12193 PMC8360643

[4] Sugano K (2015) Screening of gastric cancer in Asia. Best Pract Res Clin Gastroenterol 29(6):895–905 26651251 10.1016/j.bpg.2015.09.013

[5] Taieb J, Moehler M, Boku N, Ajani JA, Yañez Ruiz E, Ryu MH, Guenther S, Chand V, Bang YJ (2018) Evolution of checkpoint inhibitors for the treatment of metastatic gastric cancers: current status and future perspectives. Cancer Treat Rev 66:104–113 29730461 10.1016/j.ctrv.2018.04.004

[6] Wagner AD, Syn NL, Moehler M, Grothe W, Yong WP, Tai BC, Ho J, Unverzagt S (2017) Chemotherapy for advanced gastric cancer. Cochrane Database Syst Rev 8(8):40–64 10.1002/14651858.CD004064.pub4 PMC6483552 28850174

[7] Bang YJ, Van Cutsem E, Feyereislova A, Chung HC, Shen L, Sawaki A, Lordick F, Ohtsu A, Omuro Y, Satoh T et al (2010) Trastuzumab in combination with chemotherapy versus chemotherapy alone for treatment of HER2-positive advanced gastric or gastro-oesophageal junction cancer (ToGA): a phase 3, open-label, randomised controlled trial. Lancet 376(9742):687–697 20728210 10.1016/S0140-6736(10)61121-X

[8] Wilke H, Muro K, Van Cutsem E, Oh SC, Bodoky G, Shimada Y, Hironaka S, Sugimoto N, Lipatov O, Kim TY et al (2014) Ramucirumab plus paclitaxel versus placebo plus paclitaxel in patients with previously treated advanced gastric or gastro-oesophageal junction adenocarcinoma (RAINBOW): a double-blind, randomised phase 3 trial. Lancet Oncol 15(11):1224–1235 25240821 10.1016/S1470-2045(14)70420-6

[9] Li J, Qin S, Xu J, Xiong J, Wu C, Bai Y, Liu W, Tong J, Liu Y, Xu R et al (2016) Randomized, double-blind, placebo-controlled phase iii trial of apatinib in patients with chemotherapy-refractory advanced or metastatic adenocarcinoma of the stomach or gastroesophageal junction. J Clin Oncol 34(13):1448–1454 26884585 10.1200/JCO.2015.63.5995

[10] Bagchi S, Yuan R, Engleman EG (2021) Immune checkpoint inhibitors for the treatment of cancer: clinical impact and mechanisms of response and resistance. Annu Rev Pathol 16:223–249 33197221 10.1146/annurev-pathol-042020-042741

[11] Dunn GP, Bruce AT, Ikeda H, Old LJ, Schreiber RD (2002) Cancer immunoediting: from immunosurveillance to tumor escape. Nat Immunol 3(11):991–998 12407406 10.1038/ni1102-991

[12] Marshall HT, Djamgoz MBA (2018) Immuno-oncology: emerging targets and combination therapies. Front Oncol 8:315 30191140 10.3389/fonc.2018.00315 PMC6115503

[13] Nowak AK, Lake RA, Marzo AL, Scott B, Heath WR, Collins EJ, Frelinger JA, Robinson BW (2003) Induction of tumor cell apoptosis in vivo increases tumor antigen cross-presentation, cross-priming rather than cross-tolerizing host tumor-specific CD8 T cells. J Immunol (Baltimore Md 1950) 170(10):4905–4913 10.4049/jimmunol.170.10.4905 12734333

[14] Lesterhuis WJ, Punt CJ, Hato SV, Eleveld-Trancikova D, Jansen BJ, Nierkens S, Schreibelt G, de Boer A, Van Herpen CM, Kaanders JH et al (2011) Platinum-based drugs disrupt STAT6-mediated suppression of immune responses against cancer in humans and mice. J Clin Investig 121(8):3100–3108 21765211 10.1172/JCI43656 PMC3148725

[15] Pardoll DM (2012) The blockade of immune checkpoints in cancer immunotherapy. Nat Rev Cancer 12(4):252–264 22437870 10.1038/nrc3239 PMC4856023

[16] Xu J, Jiang H, Pan Y, Gu K, Cang S, Han L, Shu Y, Li J, Zhao J, Pan H et al (2023) Sintilimab plus chemotherapy for unresectable gastric or gastroesophageal junction cancer: the orient-16 randomized clinical trial. JAMA 330(21):2064–2074 38051328 10.1001/jama.2023.19918 PMC10698618

[17] Kang YK, Boku N, Satoh T, Ryu MH, Chao Y, Kato K, Chung HC, Chen JS, Muro K, Kang WK et al (2017) Nivolumab in patients with advanced gastric or gastro-oesophageal junction cancer refractory to, or intolerant of, at least two previous chemotherapy regimens (ONO-4538-12, ATTRACTION-2): a randomised, double-blind, placebo-controlled, phase 3 trial. Lancet 390(10111):2461–2471 28993052 10.1016/S0140-6736(17)31827-5

[18] Yamashita K, Iwatsuki M, Harada K, Eto K, Hiyoshi Y, Ishimoto T, Nagai Y, Iwagami S, Miyamoto Y, Yoshida N et al (2020) Prognostic impacts of the combined positive score and the tumor proportion score for programmed death ligand-1 expression by double immunohistochemical staining in patients with advanced gastric cancer. Gastric Cancer 23(1):95–104 31451991 10.1007/s10120-019-00999-9

[19] Xie T, Zhang Z, Zhang X, Qi C, Shen L, Peng Z (2021) Appropriate PD-L1 cutoff value for gastric cancer immunotherapy: a systematic review and meta-analysis. Front Oncol 11:646355 34540656 10.3389/fonc.2021.646355 PMC8440909

[20] Apetoh L, Ladoire S, Coukos G, Ghiringhelli F (2015) Combining immunotherapy and anticancer agents: The right path to achieve cancer cure? Ann Oncol 26(9):1813–1823 25922066 10.1093/annonc/mdv209

[21] Janjigian YY, Shitara K, Moehler M, Garrido M, Salman P, Shen L, Wyrwicz L, Yamaguchi K, Skoczylas T, Campos Bragagnoli A et al (2021) First-line nivolumab plus chemotherapy versus chemotherapy alone for advanced gastric, gastro-oesophageal junction, and oesophageal adenocarcinoma (CheckMate 649): a randomised, open-label, phase 3 trial. Lancet 398(10294):27–40 34102137 10.1016/S0140-6736(21)00797-2 PMC8436782

[22] Boku N, Ryu MH, Oh DY, Oh SC, Chung HC, Lee KW, Omori T, Shitara K, Sakuramoto S, Chung IJ et al (2020) LBA7_PR Nivolumab plus chemotherapy versus chemotherapy alone in patients with previously untreated advanced or recurrent gastric/gastroesophageal junction (G/GEJ) cancer: ATTRACTION-4 (ONO-4538-37) study. Ann Oncol 31:S1192 10.1016/j.annonc.2020.08.2297

[23] Shitara K, Van Cutsem E, Bang YJ, Fuchs C, Wyrwicz L, Lee KW, Kudaba I, Garrido M, Chung HC, Lee J et al (2020) Efficacy and safety of pembrolizumab or pembrolizumab plus chemotherapy vs chemotherapy alone for patients with first-line, advanced gastric cancer: the KEYNOTE-062 phase 3 randomized clinical trial. JAMA Oncol 6(10):1571–1580 32880601 10.1001/jamaoncol.2020.3370 PMC7489405

[24] Moehler MH, Kato K, Arkenau H-T, Oh D-Y, Tabernero J, Cruz-Correa M, Wang H, Xu H, Li J, Yang S et al (2023) Rationale 305: Phase 3 study of tislelizumab plus chemotherapy vs placebo plus chemotherapy as first-line treatment (1L) of advanced gastric or gastroesophageal junction adenocarcinoma (GC/GEJC). J Clin Oncol 41(4_suppl):286–286 10.1200/JCO.2023.41.4_suppl.286

[25] Zhao JJ, Yap DWT, Chan YH, Tan BKJ, Teo CB, Syn NL, Smyth EC, Soon YY, Sundar R (2022) Low programmed death-ligand 1-expressing subgroup outcomes of first-line immune checkpoint inhibitors in gastric or esophageal adenocarcinoma. J Clin Oncol 40(4):392–402 34860570 10.1200/JCO.21.01862

[26] Guideline Recommendations for HER2 Detection in Gastric Cancer Group (2011) Guidelines for HER2 detection in gastric cancer. Chin J Pathol 40(8):553–557 22169647

[27] Hanahan D, Weinberg RA (2011) Hallmarks of cancer: the next generation. Cell 144(5):646–674 21376230 10.1016/j.cell.2011.02.013

[28] Yi M, Jiao D, Xu H, Liu Q, Zhao W, Han X, Wu K (2018) Biomarkers for predicting efficacy of PD-1/PD-L1 inhibitors. Mol Cancer 17(1):129 30139382 10.1186/s12943-018-0864-3 PMC6107958

[29] Chen L, Han X (2015) Anti-PD-1/PD-L1 therapy of human cancer: past, present, and future. J Clin Invest 125(9):3384–3391 26325035 10.1172/JCI80011 PMC4588282

[30] Aarnink A, Fumet JD, Favier L, Truntzer C, Ghiringhelli F (2020) Role of pleural and peritoneal metastasis in immune checkpoint inhibitors efficacy patients with non-small cell lung cancer: real-world data from a large cohort in France. J Cancer Res Clin Oncol 146(10):2699–2707 32474752 10.1007/s00432-020-03262-2 PMC11804642

[31] Narita YSK, Mitani S, Honda K, Masuishi T, Taniguchi H et al (2017) Peritoneum metastasis (PM) as a prognostic factor in metastatic gastric cancer (MGC) treated with anti-PD-1/PD-L1 monotherapy. J Clin Oncol 5(15_suppl):3051–3061 10.1200/JCO.2017.35.15_suppl.3051

[32] Hagi T, Kurokawa Y, Kawabata R, Omori T, Matsuyama J, Fujitani K, Hirao M, Akamaru Y, Takahashi T, Yamasaki M et al (2020) Multicentre biomarker cohort study on the efficacy of nivolumab treatment for gastric cancer. Br J Cancer 123(6):965–972 32616848 10.1038/s41416-020-0975-7 PMC7492241

[33] Kang YK, Morita S, Satoh T, Ryu MH, Chao Y, Kato K, Chung HC, Chen JS, Muro K, Kang WK et al (2022) Exploration of predictors of benefit from nivolumab monotherapy for patients with pretreated advanced gastric and gastroesophageal junction cancer: post hoc subanalysis from the ATTRACTION-2 study. Gastric Cancer 25(1):207–217 34480657 10.1007/s10120-021-01230-4 PMC8732926

[34] Kawazoe A, Shitara K, Kuboki Y, Bando H, Kojima T, Yoshino T, Ohtsu A, Ochiai A, Togashi Y, Nishikawa H et al (2019) Clinicopathological features of 22C3 PD-L1 expression with mismatch repair, Epstein–Barr virus status, and cancer genome alterations in metastatic gastric cancer. Gastric Cancer 22(1):69–76 29859006 10.1007/s10120-018-0843-9

[35] Kanda M, Kodera Y (2016) Molecular mechanisms of peritoneal dissemination in gastric cancer. World J Gastroenterol 22(30):6829–6840 27570420 10.3748/wjg.v22.i30.6829 PMC4974582

[36] West H, McCleod M, Hussein M, Morabito A, Rittmeyer A, Conter HJ, Kopp HG, Daniel D, McCune S, Mekhail T et al (2019) Atezolizumab in combination with carboplatin plus nab-paclitaxel chemotherapy compared with chemotherapy alone as first-line treatment for metastatic non-squamous non-small-cell lung cancer (IMpower130): a multicentre, randomised, open-label, phase 3 trial. Lancet Oncol 20(7):924–937 31122901 10.1016/S1470-2045(19)30167-6

[37] Tumeh PC, Hellmann MD, Hamid O, Tsai KK, Loo KL, Gubens MA, Rosenblum M, Harview CL, Taube JM, Handley N et al (2017) Liver metastasis and treatment outcome with anti-pd-1 monoclonal antibody in patients with melanoma and NSCLC. Cancer Immunol Res 5(5):417–424 28411193 10.1158/2326-6066.CIR-16-0325 PMC5749922

[38] Kim SK, Kim HJ, Park JL, Heo H, Kim SY, Lee SI, Song KS, Kim WH, Kim YS (2020) Identification of a molecular signature of prognostic subtypes in diffuse-type gastric cancer. Gastric Cancer 23(3):473–482 31773340 10.1007/s10120-019-01029-4 PMC7165151

[39] Fukamachi H, Kim SK, Koh J, Lee HS, Sasaki Y, Yamashita K, Nishikawaji T, Shimada S, Akiyama Y, Byeon SJ et al (2019) A subset of diffuse-type gastric cancer is susceptible to mTOR inhibitors and checkpoint inhibitors. J Exp Clin Cancer Res 38(1):127 30866995 10.1186/s13046-019-1121-3 PMC6416873

[40] Qiu MZ, Cai MY, Zhang DS, Wang ZQ, Wang DS, Li YH, Xu RH (2013) Clinicopathological characteristics and prognostic analysis of Lauren classification in gastric adenocarcinoma in China. J Transl Med 11:58 23497313 10.1186/1479-5876-11-58 PMC3600019

[41] Cancer Genome Atlas Research N: Comprehensive molecular characterization of gastric adenocarcinoma. *Nature* 2014, 513(7517):202–209 10.1038/nature13480 PMC4170219 25079317

[42] Chen Y, Zhou Q, Wang H, Zhuo W, Ding Y, Lu J, Wu G, Xu N, Teng L (2020) Predicting peritoneal dissemination of gastric cancer in the era of precision medicine: molecular characterization and biomarkers. Cancers (Basel) 12(8):2236 32785164 10.3390/cancers12082236 PMC7547377

[43] Mishima S, Kawazoe A, Nakamura Y, Sasaki A, Kotani D, Kuboki Y, Bando H, Kojima T, Doi T, Ohtsu A et al (2019) Clinicopathological and molecular features of responders to nivolumab for patients with advanced gastric cancer. J Immunother Cancer 7(1):24 30704511 10.1186/s40425-019-0514-3 PMC6357506

[44] Shitara K, Özgüroğlu M, Bang YJ, Di Bartolomeo M, Mandalà M, Ryu MH, Fornaro L, Olesiński T, Caglevic C, Chung HC et al (2018) Pembrolizumab versus paclitaxel for previously treated, advanced gastric or gastro-oesophageal junction cancer (KEYNOTE-061): a randomised, open-label, controlled, phase 3 trial. Lancet 392(10142):123–133 29880231 10.1016/S0140-6736(18)31257-1

[45] Yu H, Boyle TA, Zhou C, Rimm DL, Hirsch FR (2016) PD-L1 expression in lung cancer. J Thorac Oncol 11(7):964–975 27117833 10.1016/j.jtho.2016.04.014 PMC5353357

[46] Müller P, Kreuzaler M, Khan T, Thommen DS, Martin K, Glatz K, Savic S, Harbeck N, Nitz U, Gluz O et al (2015) Trastuzumab emtansine (T-DM1) renders HER2+ breast cancer highly susceptible to CTLA-4/PD-1 blockade. Sci Transl Med 7(315):ra188 10.1126/scitranslmed.aac4925 26606967

[47] Chaganty BKR, Qiu S, Gest A, Lu Y, Ivan C, Calin GA, Weiner LM, Fan Z (2018) Trastuzumab upregulates PD-L1 as a potential mechanism of trastuzumab resistance through engagement of immune effector cells and stimulation of IFNγ secretion. Cancer Lett 430:47–56 29746929 10.1016/j.canlet.2018.05.009 PMC6004098

[48] Janjigian YY, Maron SB, Chatila WK, Millang B, Chavan SS, Alterman C, Chou JF, Segal MF, Simmons MZ, Momtaz P et al (2020) First-line pembrolizumab and trastuzumab in HER2-positive oesophageal, gastric, or gastro-oesophageal junction cancer: an open-label, single-arm, phase 2 trial. Lancet Oncol 21(6):821–831 32437664 10.1016/S1470-2045(20)30169-8 PMC8229851

[49] Rha SYLC, Kim H (2020) Targeting HER2 in combination with anti-PD-1 and chemotherapy confers a significant tumor shrinkage of gastric cancer: a multi-institutional Phase Ib/II trial of first-line triplet regimen (pembrolizumab, trastuzumab, chemotherapy) for HER2 positive advanced gastric cancer (AGC). J Clin Oncol 38(Suppl. 4):3081 10.1200/JCO.2020.38.15_suppl.3081

[50] Janjigian YY, Kawazoe A, Yañez P, Li N, Lonardi S, Kolesnik O, Barajas O, Bai Y, Shen L, Tang Y et al (2021) The KEYNOTE-811 trial of dual PD-1 and HER2 blockade in HER2-positive gastric cancer. Nature 600(7890):727–730 34912120 10.1038/s41586-021-04161-3 PMC8959470

[51] Diem S, Schmid S, Krapf M, Flatz L, Born D, Jochum W, Templeton AJ, Früh M (2017) Neutrophil-to-lymphocyte ratio (NLR) and platelet-to-lymphocyte ratio (PLR) as prognostic markers in patients with non-small cell lung cancer (NSCLC) treated with nivolumab. Lung Cancer 111:176–181 28838390 10.1016/j.lungcan.2017.07.024

[52] Fan X, Wang D, Zhang W, Liu J, Liu C, Li Q, Ma Z, Li H, Guan X, Bai Y et al (2021) Inflammatory markers predict survival in patients with advanced gastric and colorectal cancers receiving anti–PD-1 therapy. Front Cell Dev Biol 15(9):638312 10.3389/fcell.2021.638312 PMC8005614 33791296

[53] Wan M, Ding Y, Mao C, Ma X, Li N, Xiao C, Qian J, Jiang H, Zheng Y, Wu L et al (2022) Association of inflammatory markers with survival in patients with advanced gastric cancer treated with immune checkpoint inhibitors combined with chemotherapy as first line treatment. Front Oncol 12:1029960 36387183 10.3389/fonc.2022.1029960 PMC9650180

[54] Oster P, Vaillant L, Riva E, McMillan B, Begka C, Truntzer C, Richard C, Leblond MM, Messaoudene M, Machremi E et al (2022) *Helicobacter pylori* infection has a detrimental impact on the efficacy of cancer immunotherapies. Gut 71(3):457–466 34253574 10.1136/gutjnl-2020-323392 PMC8862014

