# Supplementary material for: Clinical outcomes and biomarker exploration of first-line PD-1 inhibitors plus chemotherapy in patients with low PD-L1-expressing of gastric or gastroesophageal junction adenocarcinoma
Source: Cancer Immunol Immunother. 2024 Jun 4;73(8):144. doi: 10.1007/s00262-024-03721-6 (PMC11150231; doi:10.1007/s00262-024-03721-6)
Supplement: Supplementary file 1 — Supplementary file1 (DOCX 25 KB) [file 262_2024_3721_MOESM1_ESM.docx]

Supplementary Table S1. Baseline characteristics of all enrolled patients

| Characteristics | Total (*n* = 345) |
| --- | --- |
| Age (years), median (range) | 57 (21-82) |
| Sex |  |
| Male | 215 (62.3%) |
| Female | 130 (37.7%) |
| ECOG PS |  |
| 0 | 255 (73.9%) |
| 1 | 84 (24.4%) |
| 2 | 6 (1.7%) |
| Histology |  |
| Diffuse | 129 (37.4%) |
| Intestinal | 104 (30.1%) |
| Mixed | 59 (17.1%) |
| Not available | 53 (15.4%) |
| BMI (kg/m^2^) |  |
| $<$18.5 | 54 (15.7%) |
| 18.5-23.9 | 222 (64.3%) |
| $\geq$24 | 69 (20.0%) |
| Primary tumor location |  |
| Gastric cancer | 308 (89.3%) |
| Gastroesophageal junction cancer | 37 (10.7%) |
| Differentiation |  |
| High or middle differentiation | 48 (13.9%) |
| Low differentiation | 297 (86.1%) |
| *Helicobacter pylori* infection |  |
| Yes | 97 (28.1%) |
| No | 51 (14.8%) |
| Not available | 197 (57.1%) |
| PD-L1 CPS |  |
| $<$1 | 61 (17.7%) |
| $\geq$1 | 110 (31.9%) |
| $<$5 | 116 (33.6%) |
| $\geq$5 | 55 (16.0%) |
| Not available | 174 (50.4%) |
| MMR status |  |
| P-MMR / MSS | 261 (75.7%) |
| D-MMR / MSI-H | 10 (2.9%) |
| Not available | 74 (21.4%) |
| HER2 |  |
| Positive | 58 (16.8%) |
| Negative | 275 (79.7%) |
| Not available | 12 (3.5%) |
| EBV |  |
| Positive | 19 (5.5%) |
| Negative | 203 (58.8%) |
| Not available | 123 (35.7%) |
| TMB (mutations/Mb) |  |
| TMB$\geq$10 | 4 (1.1%) |
| TMB$<$10 | 63 (18.3%) |
| Not available | 278 (80.6%) |
| Previous gastrectomy |  |
| Yes | 100 (29.0%) |
| No | 245 (71.0%) |
| Site of metastasis |  |
| Lymph node | 189 (54.8%) |
| Peritoneum | 170 (49.3%) |
| Liver | 119 (34.5%) |
| Ovary | 45 (13.0%) |
| Lung | 33 (9.6%) |
| Bone | 23 (6.7%) |
| Number of metastatic sites |  |
| $\leq$1 | 160 (46.4%) |
| $\geq$2 | 185 (53.6%) |
| Chemotherapy regimen |  |
| SOX | 154 (44.6%) |
| XELOX | 141 (40.9%) |
| FOLFOX | 49 (14.2%) |
| Capecitabine | 1 (0.3%) |

ECOG PS, Eastern Cooperative Oncology Group performance status; BMI, body mass index; PD-L1, programmed death-ligand 1; CPS, combined positive score; MMR, mismatch repair; P-MMR, MMR-proficient; D-MMR, MMR-deficient; MSI-H, microsatellite instability-high; MSS, microsatellite stable; HER2, human epidermal growth factor receptor 2; EBV, Epstein-Barr virus; TMB, tumor mutational burden.
