# Supplementary material for: Clinical outcomes and biomarker exploration of first-line PD-1 inhibitors plus chemotherapy in patients with low PD-L1-expressing of gastric or gastroesophageal junction adenocarcinoma
Source: Cancer Immunol Immunother. 2024 Jun 4;73(8):144. doi: 10.1007/s00262-024-03721-6 (PMC11150231; doi:10.1007/s00262-024-03721-6)
Supplement: Supplementary file 2 — Supplementary file2 (DOCX 26 KB) [file 262_2024_3721_MOESM2_ESM.docx]

Supplementary Table S2. Comparison of baseline characteristics between patients with PD-L1 CPS<5 and CPS≥5

| Characteristics | PD-L1 CPS<5 (*n* = 116) | PD-L1 CPS≥5  (*n* = 55) | P value |
| --- | --- | --- | --- |
| Age (years), median (range) | 57 (25-75) | 54 (22-82) | 0.665 |
| Sex |  |  | 0.843 |
| Male | 72 (62.1%) | 35 (63.6%) |  |
| Female | 44 (37.9%) | 20 (36.4%) |  |
| ECOG PS |  |  | 1.000 |
| 0 | 85 (73.3%) | 41 (74.5%) |  |
| 1 | 29 (25.0%) | 14 (25.5%) |  |
| 2 | 2 (1.7%) | 0 (0.0%) |  |
| Histology |  |  | 0.225 |
| Diffuse | 47 (40.5%) | 22 (40.0%) |  |
| Intestinal | 42 (36.2%) | 13 (23.6%) |  |
| Mixed | 15 (12.9%) | 11 (20.0%) |  |
| Not available | 12 (10.4%) | 9 (16.4%) |  |
| BMI (kg/m^2^) |  |  | 0.514 |
| $<$18.5 | 15 (12.9%) | 8 (14.5%) |  |
| 18.5-23.9 | 73 (62.9%) | 38 (69.1%) |  |
| $\geq$24 | 28 (24.2%) | 9 (16.4%) |  |
| Primary tumor location |  |  | 0.069 |
| Gastric cancer | 106 (91.4%) | 45 (81.8%) |  |
| Gastroesophageal junction cancer | 10 (8.6%) | 10 (18.2%) |  |
| Differentiation |  |  | 0.281 |
| High or middle differentiation | 20 (17.2%) | 6 (10.9%) |  |
| Low differentiation | 96 (82.8%) | 49 (89.1%) |  |
| *Helicobacter pylori* infection |  |  | 0.149 |
| Yes | 35 (30.2%) | 18 (32.7%) |  |
| No | 22 (19.0%) | 5 (9.1%) |  |
| Not available | 59 (50.8%) | 32 (58.2%) |  |
| MMR status |  |  | 0.331 |
| P-MMR / MSS | 97 (83.6%) | 47 (85.5%) |  |
| D-MMR / MSI-H | 0 (0.0%) | 1 (1.8%) |  |
| Not available | 19 (16.4%) | 7 (12.7%) |  |
| HER2 |  |  | 0.547 |
| Positive | 21 (18.1%) | 8 (14.5%) |  |
| Negative | 94 (81.0%) | 47 (85.5%) |  |
| Not available | 1 (0.9%) | 0 (0.0%) |  |
| EBV |  |  | <0.001 |
| Positive | 1 (0.9%) | 10 (18.2%) |  |
| Negative | 91 (78.4%) | 27 (49.1%) |  |
| Not available | 24 (20.7%) | 18 (32.7%) |  |
| TMB (mutations/Mb) |  |  | 0.442 |
| TMB$\geq$10 | 1 (0.9%) | 1 (1.8%) |  |
| TMB$<$10 | 32 (27.6%) | 10 (18.2%) |  |
| Not available | 83 (71.5%) | 44 (80.0%) |  |
| Previous gastrectomy |  |  | 0.238 |
| Yes | 33 (28.5%) | 11 (20.0%) |  |
| No | 83 (71.5%) | 44 (80.0%) |  |
| Site of metastasis |  |  |  |
| Peritoneum | 67 (57.8%) | 20 (36.4%) | 0.009 |
| Lymph node | 56 (48.3%) | 36 (65.5%) | 0.035 |
| Liver | 40 (34.5%) | 22 (40.0%) | 0.483 |
| Ovary | 17 (14.7%) | 5 (9.1%) | 0.287 |
| Lung | 16 (13.8%) | 5 (9.1%) | 0.382 |
| Number of metastatic sites |  |  | 0.846 |
| $\leq$1 | 53 (45.7%) | 26 (47.3%) |  |
| $\geq$2 | 63 (54.3%) | 29 (52.7%) |  |

ECOG PS, Eastern Cooperative Oncology Group performance status; BMI, body mass index; PD-L1, programmed death-ligand 1; CPS, combined positive score; MMR, mismatch repair; P-MMR, MMR-proficient; D-MMR, MMR-deficient; MSI-H, microsatellite instability-high; MSS, microsatellite stable; HER2, human epidermal growth factor receptor 2; EBV, Epstein-Barr virus; TMB, tumor mutational burden.
