# Supplementary material for: Clinical outcomes and biomarker exploration of first-line PD-1 inhibitors plus chemotherapy in patients with low PD-L1-expressing of gastric or gastroesophageal junction adenocarcinoma
Source: Cancer Immunol Immunother. 2024 Jun 4;73(8):144. doi: 10.1007/s00262-024-03721-6 (PMC11150231; doi:10.1007/s00262-024-03721-6)
Supplement: Supplementary file 3 — Supplementary file3 (DOCX 29 KB) [file 262_2024_3721_MOESM3_ESM.docx]

Supplementary Table S3. Clinicopathologic features of responders and non-responders in the total population

| Characteristics |  | Responder  *N* = 172 | Non-responder  *N* = 118 | ORR | *P* value |
| --- | --- | --- | --- | --- | --- |
| Age | $<$60 | 88 | 71 | 55.3% | 0.130 |
|  | $\geq$60 | 84 | 47 | 64.1% |  |
| Sex | Male | 120 | 68 | 63.8% | 0.033 |
|  | Female | 52 | 50 | 51.0% |  |
| BMI | $<$18.5 | 25 | 16 | 61.0% | 0.719 |
|  | 18.5-23.9 | 109 | 80 | 57.7% |  |
|  | $\geq$24 | 38 | 22 | 63.3% |  |
| Histology | Diffuse | 49 | 53 | 48.0% | 0.001 |
|  | Non-diffuse | 98 | 45 | 68.5% |  |
| Primary tumor location | Gastric cancer | 151 | 109 | 58.1% | 0.208 |
|  | Gastroesophageal junction cancer | 21 | 9 | 70.0% |  |
| Differentiation | High or middle differentiation | 31 | 16 | 66.0% | 0.311 |
|  | Low differentiation | 141 | 102 | 58.0% |  |
| Disease status | Synchronous metastasis | 150 | 87 | 63.3% | 0.004 |
|  | Metachronous metastasis | 22 | 31 | 41.5% |  |
| ECOG PS | 0 | 135 | 82 | 62.2% | 0.083 |
|  | $\geq$1 | 37 | 36 | 50.7% |  |
| Site of metastasis |  |  |  |  |  |
| Peritoneum | Yes | 52 | 72 | 41.9% | $<$0.001 |
|  | No | 120 | 46 | 72.3% |  |
| Liver | Yes | 80 | 39 | 67.2% | 0.022 |
|  | No | 92 | 79 | 53.8% |  |
| Lymph node | Yes | 121 | 62 | 66.1% | 0.002 |
|  | No | 51 | 56 | 47.7% |  |
| Ovary | Yes | 13 | 21 | 38.2% | 0.069 |
|  | No | 39 | 29 | 57.4% |  |
| Number of metastatic sites | $\leq$1 | 69 | 51 | 57.5% | 0.598 |
|  | $\geq$2 | 103 | 67 | 60.6% |  |
| MMR status | P-MMR/MSS | 117 | 98 | 54.4% | 1.000 |
|  | D-MMR/MSI-H | 4 | 4 | 50.0% |  |
| HER2 | Positive | 42 | 11 | 79.2% | 0.001 |
|  | Negative | 125 | 101 | 55.3% |  |
| EBV | Positive | 11 | 3 | 78.6% | 0.065 |
|  | Negative | 88 | 78 | 53.0% |  |
| PD-L1 CPS | $<$1 | 23 | 24 | 48.9% | 0.135 |
|  | $\geq$1 | 59 | 36 | 62.1% |  |
|  | $<$5 | 48 | 46 | 51.1% | 0.024 |
|  | $\geq$5 | 34 | 14 | 70.8% |  |
|  | $<$10 | 65 | 55 | 54.2% | 0.059 |
|  | $\geq$10 | 18 | 6 | 75.0% |  |
| TMB | $<$10 | 22 | 31 | 41.5% | 0.571 |
|  | $\geq$10 | 2 | 1 | 66.7% |  |
| Type of PD-1 inhibitor | Nivolumab | 27 | 26 | 50.9% | 0.188 |
|  | Pembrolizumab | 7 | 5 | 58.3% |  |
|  | Toripalimab | 23 | 13 | 63.9% |  |
|  | Sintilimab | 94 | 51 | 64.8% |  |
|  | Tislelizumab | 21 | 23 | 47.7% |  |
| Baseline NLR | $<$3 | 86 | 66 | 56.6% | 0.320 |
|  | $\geq$3 | 86 | 52 | 62.3% |  |
| Baseline MLR | $<$0.31 | 77 | 60 | 56.2% | 0.308 |
|  | $\geq$0.31 | 95 | 58 | 62.1% |  |
| Baseline PLR | $<$188 | 87 | 55 | 61.3% | 0.506 |
|  | $\geq$188 | 85 | 63 | 57.4% |  |
| *Helicobacter pylori* infection | Yes | 50 | 33 | 60.2% | 0.485 |
|  | No | 22 | 19 | 53.7% |  |

ORR, objective response rate; ECOG PS, Eastern Cooperative Oncology Group performance status; BMI, body mass index; PD-1, programmed death 1; PD-L1, programmed death-ligand 1; CPS, combined positive score; MMR, mismatch repair; P-MMR, MMR-proficient; D-MMR, MMR-deficient; MSI-H, microsatellite instability-high; MSS, microsatellite stable; HER2, human epidermal growth factor receptor 2; EBV, Epstein-Barr virus; TMB, tumor mutational burden; NLR, neutrophil-to-lymphocyte ratio; MLR, monocyte-to-lymphocyte ratio; PLR, platelet-to-lymphocyte ratio.
