# Supplementary material for: Clinical outcomes and biomarker exploration of first-line PD-1 inhibitors plus chemotherapy in patients with low PD-L1-expressing of gastric or gastroesophageal junction adenocarcinoma
Source: Cancer Immunol Immunother. 2024 Jun 4;73(8):144. doi: 10.1007/s00262-024-03721-6 (PMC11150231; doi:10.1007/s00262-024-03721-6)
Supplement: Supplementary file 4 — Supplementary file3 (DOCX 26 KB) [file 262_2024_3721_MOESM4_ESM.docx]

Supplementary Table S4. Univariate and multivariate analyses for progression-free survival in the total population

| Variables |  | Univariate analysis | | Multivariate analysis | |
| --- | --- | --- | --- | --- | --- |
|  |  | HR (95% CI) | *P* value | HR (95% CI) | *P* Value |
| Age | $\geq$60 vs$<$60 | 0.79(0.61,1.02) | 0.070 | - | - |
| Sex | Male vs female | 0.72(0.56-0.93) | 0.013 | 0.83(0.52-1.33) | 0.450 |
| BMI | $<$18.5 | Reference |  |  |  |
|  | 18.5-23.9 | 0.94(0.66-1.33) | 0.709 | - | - |
|  | $\geq$24 | 0.85(0.56-1.29) | 0.440 | - | - |
| ECOG PS | 0 vs$\geq$1 | 0.83(0.63-1.10) | 0.193 | - | - |
| Primary tumor location | GEJC vs GC | 0.65(0.42-1.02) | 0.061 | 0.86(0.34-2.18) | 0.750 |
| Histology | Diffuse vs non-diffuse | 1.64(1.24-2.16) | < 0.001 | 1.88(1.11-3.19) | 0.018 |
| Differentiation | High or middle differentiation vs low differentiation | 0.68(0.46-1.03) | 0.068 | 1.37(0.68-2.77) | 0.382 |
| Disease status | Synchronous metastasis  vs metachronous metastasis | 0.99(0.71-1.38) | 0.934 | - | - |
| Number of metastatic sites | $\leq$1 vs$\geq$2 | 0.64(0.50-0.83) | 0.001 | 0.65(0.42-1.01) | 0.054 |
| Site of metastasis | Peritoneum | 1.96(1.51-2.54) | < 0.001 | 1.88(1.11-3.17) | 0.018 |
|  | With ascites | 1.89(1.34-2.68) | < 0.001 | - | - |
|  | Lymph node | 0.85(0.66,1.10) | 0.207 | - | - |
|  | Liver | 1.17(0.90-1.53) | 0.240 | - | - |
|  | Ovary | 0.99(0.65-1.51) | 0.967 | - | - |
| PD-L1 CPS | $\geq$1 vs$<$1 | 0.78(0.54-1.13) | 0.194 | - | - |
|  | $\geq$5 vs$<$5 | 0.58(0.38-0.87) | 0.008 | 0.43(0.23-0.81) | 0.009 |
|  | $\geq$10 vs$<$10 | 0.89(0.54-1.46) | 0.639 | - | - |
| HER2 | Positive vs negative | 0.54(0.37-0.79) | 0.001 | 0.46(0.21-0.99) | 0.048 |
| EBV | Positive vs negative | 0.77(0.43-1.39) | 0.390 | 0.93(0.28-3.10) | 0.907 |
| MMR status | D-MMR/MSI-H vs P-MMR/ MSS | 0.34(0.13-0.95) | 0.040 | - | - |
| TMB | $\geq$10 vs $<$10 | 0.11(0.02-0.83) | 0.032 | - | - |
| Baseline NLR | $\geq$3 vs$<$3 | 1.18(0.91-1.52) | 0.215 | - | - |
| Baseline MLR | $\geq$0.31 vs$<$0.31 | 1.02(0.79-1.31) | 0.906 | - | - |
| Baseline PLR | $\geq$188 vs$<$188 | 1.38(1.07-1.78) | 0.013 | 1.75(1.10-2.78) | 0.018 |
| *Helicobacter pylori* infection | Yes vs no | 1.27(0.85-1.90) | 0.247 | - | - |

HR, hazard ratio; CI, confidence interval; ECOG PS, Eastern Cooperative Oncology Group performance status; BMI, body mass index; GC, gastric cancer; GEJC, gastroesophageal junction cancer; PD-L1, programmed death-ligand 1; CPS, combined positive score; MMR, mismatch repair; P-MMR, MMR-proficient; D-MMR, MMR-deficient; MSI-H, microsatellite instability-high; MSS, microsatellite stable; HER2, human epidermal growth factor receptor 2; EBV, Epstein-Barr virus; TMB, tumor mutational burden; NLR, neutrophil-to-lymphocyte ratio; MLR, monocyte-to-lymphocyte ratio; PLR, platelet-to-lymphocyte ratio.
