# Supplementary material for: Clinical outcomes and biomarker exploration of first-line PD-1 inhibitors plus chemotherapy in patients with low PD-L1-expressing of gastric or gastroesophageal junction adenocarcinoma
Source: Cancer Immunol Immunother. 2024 Jun 4;73(8):144. doi: 10.1007/s00262-024-03721-6 (PMC11150231; doi:10.1007/s00262-024-03721-6)
Supplement: Supplementary file 5 — Supplementary file3 (DOCX 28 KB) [file 262_2024_3721_MOESM5_ESM.docx]

Supplementary Table S5. Univariate and multivariate analyses for overall survival in the total population

| Variables |  | Univariate analysis | | Multivariate analysis | |
| --- | --- | --- | --- | --- | --- |
|  |  | HR (95% CI) | *P* value | HR (95% CI) | *P* value |
| Age | $\geq$60 vs$<$60 | 1.00(0.69,1.45) | 0.986 | - | - |
| Sex | Male vs female | 0.95(0.65-1.39) | 0.805 | - | - |
| BMI | $<$18.5 | Reference |  |  |  |
|  | 18.5-23.9 | 1.32(0.76-2.30) | 0.327 | - | - |
|  | $\geq$24 | 1.12(0.58-2.13) | 0.743 | - | - |
| ECOG PS | 0 vs$\geq$1 | 0.68(0.46-0.99) | 0.042 | 0.83(0.47-1.48) | 0.524 |
| Primary tumor location | GEJC vs GC | 0.46(0.21-0.98) | 0.044 | 0.64(0.18-2.32) | 0.499 |
| Histology | Diffuse vs non-diffuse | 1.84(1.24-2.71) | 0.002 | 1.65(0.89-3.08) | 0.115 |
| Differentiation | High or middle differentiation vs low differentiation | 0.62(0.33-1.15) | 0.126 | - | - |
| Disease status | Synchronous metastasis  vs metachronous metastasis | 0.92(0.58-1.46) | 0.711 | - | - |
| Number of metastatic sites | $\leq$1 vs$\geq$2 | 0.67(0.46-0.97) | 0.033 | 1.08(0.62-1.90) | 0.785 |
| Site of metastasis | Peritoneum | 1.86(1.27-2.71) | 0.001 | 2.17(1.12-4.20) | 0.022 |
|  | With ascites | 2.27(1.36-3.78) | 0.002 | - | - |
|  | Lymph node | 1.20(0.82,1.75) | 0.349 | - | - |
|  | Liver | 0.97(0.65-1.43) | 0.858 | - | - |
|  | Ovary | 0.51(0.25-1.01) | 0.053 | - | - |
| PD-L1 CPS | $\geq$1 vs$<$1 | 0.80(0.48-1.34) | 0.398 | - | - |
|  | $\geq$5 vs$<$5 | 0.63(0.35-1.15) | 0.134 | 0.72(0.37-1.40) | 0.332 |
|  | $\geq$10 vs$<$10 | 0.78(0.35-1.71) | 0.532 | - | - |
| HER2 | Positive vs negative | 0.78(0.35-1.04) | 0.067 | 0.84(0.33-2.15) | 0.708 |
| EBV | Positive vs negative | 0.66(0.27-1.63) | 0.368 | - | - |
| MMR status | D-MMR/MSI-H vs P-MMR/ MSS | 0.42(0.10-1.75) | 0.235 | - | - |
| TMB | $\geq$10 vs $<$10 | 0.19(0.02-1.55) | 0.121 | - | - |
| Baseline NLR | $\geq$3 vs$<$3 | 1.82(1.26-2.63) | 0.001 | 1.74(0.81-3.77) | 0.157 |
| Baseline MLR | $\geq$0.31 vs$<$0.31 | 1.78(1.22-2.58) | 0.003 | 1.25(0.58-2.69) | 0.573 |
| Baseline PLR | $\geq$188 vs$<$188 | 1.30(0.90-1.87) | 0.167 | - | - |
| *Helicobacter pylori* infection | Yes vs no | 0.91(0.55-1.49) | 0.697 | - | - |

HR, hazard ratio; CI, confidence interval; ECOG PS, Eastern Cooperative Oncology Group performance status; BMI, body mass index; GC, gastric cancer; GEJC, gastroesophageal junction cancer; PD-1, programmed death-1; PD-L1, programmed death-ligand 1; CPS, combined positive score; MMR, mismatch repair; P-MMR, MMR-proficient; D-MMR, MMR-deficient; MSI-H, microsatellite instability-high; MSS, microsatellite stable; HER2, human epidermal growth factor receptor 2; EBV, Epstein-Barr virus; TMB, tumor mutational burden; NLR, neutrophil-to-lymphocyte ratio; MLR, monocyte-to-lymphocyte ratio; PLR, platelet-to-lymphocyte ratio.
